# Revolutionizing Hypoglycemia Management in Long-Term Care: Lessons Learned From a Pilot Quality Improvement Initiative Using Continuous Glucose Monitoring

**DOI:** 10.2196/73485

**Published:** 2025-10-20

**Authors:** Denis O'Donnell, Sue Burns, Shirley Drever, Lisa Quesnelle, Benjamin Yuen

**Affiliations:** 1 CareRx Corporation, 320 Bay St. Suite 1200, Toronto, ON, M5H 4A6, Canada, 1 647-282-2736, 1 416-927-8405; 2 Sienna Senior Living, Markham, ON, Canada; 3 Extendicare (Canada), Markham, ON, Canada

**Keywords:** hypoglycemia, diabetes, long-term care, continuous glucose monitoring, detection, nursing

## Abstract

Despite efforts to raise glycemic targets and reduce modifiable risk factors, hypoglycemia continues to impact a large number of long-term care (LTC) residents living with diabetes mellitus and remains one of the leading causes of hospitalization in this cohort. Effective, sustainable practice strategies to monitor and maintain glycemic control in LTC are lacking. We describe the stepwise approach used by 2 LTC homes that switched from traditional fingerstick testing to a continuous glucose monitoring (CGM) system as part of a quality improvement initiative to reduce nursing workload and address hypoglycemia. This was an exploratory pilot project. A working group was established at each of the 2 participating LTC homes, including representatives from management and direct care staff. Kickoff meetings were held with key direct care staff to discuss the limitations of current monitoring practices and potential solutions. The following interventions were agreed upon and implemented by the working groups: (1) the initiation of structured glucose monitoring for residents using CGM (FreeStyle Libre 2), requiring scanning of sensors 4 times per day; (2) provision of staff education and training on CGM by a diabetes expert; and (3) scheduling of interdisciplinary rounds as needed to optimize diabetes management. System changes were gradually introduced in a stepwise manner over a 3-month period (intervention phase), during which the LTC homes progressed from traditional fingerstick testing to point-of-care sensor readings and then to full use of the CGM software platform. Hypoglycemia was defined as a glucose reading of ≤4 mmol/L. Glucose readings were collected from 38 residents living with diabetes mellitus and receiving insulin in the 6 months before the start of the intervention phase (baseline evaluation) and in the 6 months after the end of the intervention phase (post-launch evaluation). All hypoglycemic readings detected by a sensor at a point-of-care test were validated using a fingerstick test. Nursing workload associated with glucose testing was assessed in an anonymous survey of nursing staff at baseline and after the launch. The approach resulted in a 40% reduction in nursing time required to complete a glucose reading (from 5.1 min per test at baseline to 3.1 min per test at the post-launch evaluation). The frequency of glucose monitoring increased from a total of 19,438 glucose readings in the baseline evaluation to 35,971 point-of-care sensor scans in the post-launch evaluation. The number of detected hypoglycemic events increased 12-fold, from 88 in the baseline evaluation to 1049 in the post-launch evaluation. Hypoglycemic events continue to impact a large number of LTC residents living with diabetes mellitus. CGM can improve the detection of hypoglycemic events while decreasing nursing workload. A gradual transition to CGM can help overcome underlying barriers and concerns and ensure a sustainable approach.

## Introduction

### Impact of Hypoglycemic Events in Long-Term Care

Hypoglycemia affects a large number of long-term care (LTC) residents living with diabetes mellitus [1] and is recognized as one of several leading causes of potentially avoidable hospitalizations in this cohort [2]. Based on an analysis of US hospital data, hypoglycemia is diagnosed in roughly 6% (6609/106,602) of hospital admissions among LTC residents living with diabetes [3]. Results of another US-based study suggest that, on average, 1 in 4 LTC residents living with type 2 diabetes mellitus experience severe hypoglycemia (<3 mmol/L) each year [4]. Severe hypoglycemia has been shown to be approximately 2-fold more prevalent in LTC residents with dementia than in those without dementia [5]. Other independent risk factors for hypoglycemia include increasing age, frailty, and reduced renal function [6,7]. Modifiable risk factors include erratic meal consumption and the administration of sulfonylureas and/or insulin [8]. As awareness of the considerable impact of hypoglycemia on resident health increases, current practice guidelines for older adults with diabetes mellitus have revised glycemic targets, including raising glycated hemoglobin (HbA_1c_) levels, to help prevent hypoglycemic events [6,9].

### Barriers to Preventing Hypoglycemic Events in LTC

Despite the revised glycemic targets, there remains continued reluctance among prescribers to de-intensify hypoglycemic regimens [10]. Recent studies have found that up to 40% of residents met the criteria for “overtreatment” or “potential overtreatment” with the continued use of potentially hypoglycemia-inducing drugs such as insulin or a sulfonylurea [11-13]. Following an emergency department visit or hospitalization for hypoglycemia, fewer than half of all patients had documented reductions in sulfonylurea or insulin use [14]. Reluctance to de-intensify may be due, in part, to a fear of jeopardizing diabetes control and forfeiting health benefits associated with the current medication regimen [15]. It may also reflect a need for better guidance on how to deprescribe hypoglycemic regimens [16]. Limitations inherent to current glycemic monitoring practices in the LTC setting may also contribute to hesitation among prescribers in changing treatment regimens. At present, fingerstick blood glucose testing is the preferred approach for glycemic monitoring with continuous glucose monitoring (CGM) still viewed as an emerging technology in LTC. While efforts in LTC have focused on moderating glycemic control and setting more appropriate HbA_1c_ targets, a rigorous and non-invasive approach for detecting and preventing hypoglycemia events is still lacking.

### Structured Blood Glucose Monitoring and the Prevention of Hypoglycemia

Structured blood glucose monitoring, which is characterized by a set schedule of actionable blood glucose measurements taken at specific times to guide individualized care, has been shown to improve glycemic control compared with unstructured testing [17]. Traditionally, structured blood glucose monitoring has involved either taking 5‐7 measurements daily over 1‐3 days or through “staggered” testing across a week. The current clinical practice guidelines of Diabetes Canada recommend the use of structured glucose monitoring, including the gathering of a 7-point blood glucose profile every 1‐3 months until glycemic targets are achieved. The guidelines also recommend more frequent glucose testing (4 times per day and/or overnight) when HbA_1c_ is not at target or there are episodes of hypoglycemia [18]. Unfortunately, time constraints, staffing shortages, resident discomfort, and resistance to multiple daily fingersticks are significant barriers to this intensive monitoring approach in LTC [17]. Dehydration and poor circulation, both of which are common among elderly people, may also affect the accuracy of fingerstick test results [19,20].

### Incorporating CGM Into Hypoglycemia Prevention Strategies

There is growing evidence demonstrating that CGM can serve as an effective alternative to structured blood glucose monitoring and help to reduce hypoglycemic events and glycemic variability [21,22]. CGM has the potential to provide a more comprehensive view of hypoglycemic events than fingerstick testing [23] while reducing nursing workload and improving resident quality of life. In fact, current guidelines now recommend CGM when possible as a way to reduce HbA_1c_ levels and the duration of hypoglycemia [18].

Despite these potential benefits, the adoption of this new technology in the LTC setting has been slow, with blood glucose monitoring remaining the standard approach. Concerns regarding accuracy and reliability are often cited as the primary reasons for not using the technology. These concerns may stem from outdated manufacturer warnings associated with early versions of CGM technology, which indicated that readings might be inaccurate for a few hours after a new sensor is applied due to “sensor calibration.” There is sometimes apprehension related to the clinical importance of “lag time,” the time required for glucose to diffuse across blood vessel walls into the interstitial space. Lastly, attempts to compare the performance of different glucose monitoring systems using the mean absolute relative difference (MARD) have also contributed to misgivings regarding the technology. Additional barriers in the LTC setting include the practical considerations related to accessing test results using the CGM software platform, developing fluency with CGM metrics (eg, time in range and trend arrows), and communicating results to those unfamiliar with the system. While successful incorporation of CGM into daily monitoring strategies can effectively reduce hypoglycemia as well as assist in the detection of hypoglycemic events [24], overcoming these practice barriers requires a comprehensive, stepwise implementation strategy to ensure staff are adequately trained and comfortable with the system.

### Implementation of a Hypoglycemia Prevention Strategy Using CGM in LTC Practice

Quality improvement initiatives focusing on implementing CGM in LTC can serve as useful guides for overcoming practical challenges related to everyday practice [25,26]. In October 2021, a pilot quality improvement initiative was launched at 2 LTC facilities in northern Ontario. The pilot initiative focused on a new diabetes practice model in LTC using CGM to reduce nursing workload and address hypoglycemic events. While the use of CGM successfully reduced nursing time, it also led to a 12-fold increase in the detection of hypoglycemic events, highlighting the extent of previously unrecognized episodes. This enhanced detection provided critical visibility into the problem and represented an essential first step toward driving meaningful reductions. Despite the pilot project not yet having led to reductions in the frequency of hypoglycemic events over a predetermined 6-month time period, it produced a suitable framework for overcoming major barriers to detecting hypoglycemia in LTC. The intent of this study is to describe the stepwise approach used by the LTC homes that participated in this quality improvement initiative. Learnings from this project may help other LTC homes transition smoothly and safely to a new diabetes management practice model for detecting hypoglycemic events while improving resident health and safety as well as reducing nursing workload.

## Step-Wise Approach to CGM Implementation

### Planning

Prior to commencement, the project received approval from the LTC homes’ internal research ethics board. The ARECCI Ethics Guideline Tool was used to confirm that the initiative qualified as a quality improvement project. As such, resident-level data collected during the initiative were accessed only by individuals within the circle of care. Home-level findings were de-identified and shared only in aggregate with the broader team, and only to the extent necessary to support quality improvement efforts. No additional compensation was provided to residents or staff as part of this project.

### Baseline Evaluation

In order to adhere to formal quality improvement methodology, a working group was established at each facility. The working group included representatives from both management and direct care staff who had an interest in mitigating the risk of hypoglycemic events. A baseline assessment of current diabetes management practices and the incidence of hypoglycemic events were shared with the working group. At program launch, both LTC homes were performing glucose monitoring using registered staff in accordance with each resident’s individualized care plan. This was being done predominantly through fingerstick testing. Although CGM had already been partially introduced at one of the two facilities at the time of program start-up, the CGM software was not being used and all clinical decisions were based on the point-of-care scanning of glucose readings. These were being transcribed to the electronic health record in the same way as fingerstick test results.

All residents with diabetes on insulin were considered eligible for inclusion in this study (based on Ontario requirements for coverage). At baseline, a cohort of 38 residents living with diabetes mellitus and receiving insulin were initially examined from the participating LTC homes. This represented 31% (38/121) of residents living with diabetes mellitus and 28% (38/429) of the total resident population across both facilities. A preliminary analysis included a review of 19,438 glucose readings recorded over the previous 6 months.

Nursing workload associated with fingerstick testing was assessed using the aggregate findings of the self-reported estimates of time taken to complete glucose monitoring (including the time taken to access the resident’s record, approach the resident, perform the scan, and record the data) in an anonymous paper-based survey of nursing staff, completed during mandatory staff meetings.

Clinical outcome measures were selected from a variety of available surrogate markers of health (eg, glucose readings and the presence of risk factors) and critical health events (hypoglycemia, falls, and hospitalizations) with consideration given to both relevance and sustainability. Hypoglycemic events, defined as glucose readings ≤4 mmol/L, were chosen as the most straightforward outcome measure for this pilot project.

### Implementation of the CGM System

Kickoff meetings with key direct care staff were held both to discuss limitations associated with current monitoring practices and to empower staff with some potential solutions. Issues raised at the kickoff meetings are summarized in Table 1.

**Table 1.** Problems with the current approach to glucose monitoring as discussed at the kickoff meetings along with proposed solutions.

Problem	Key concerns	Proposed solutions
Majority of point-of-care glucose test readings are not incorporated into clinical decision making	Large number of glucose readings are collected and entered into the electronic health record but not used Chronological list of glucose readings does not facilitate trend analysis Current timing of point-of-care testing does not provide a complete 24-h glycemic profile Hypoglycemia prevention is left in order to deal with more urgent health matters; also, difficulty in detecting trends with linear reporting of values	Introduction of the CGM^a^ software to provide a comprehensive glycemic profile Implementation of 4 daily scans of the sensor in order to capture a complete 24-h view of glycemic control Scheduling of diabetes medication review
Continued uncertainty as to whether sensor readings are reliable and accurate and when to double-check readings using a fingerstick blood glucose test	Unclear if newly applied sensors are reliable. Historically, staff were instructed to perform fingerstick testing for 48 h following the initial application of a FreeStyle Libre sensor Unclear when sensor readings should be validated using fingerstick testing	Discontinuation of LTC^b^ home policy requiring 48-h fingerstick monitoring following the initial application of a new sensor (with sensor scanning now possible 1 h after application) Verification using fingerstick testing only for unexpected readings and hypoglycemic events

a CGM: continuous glucose monitoring.

b LTC: long-term care.

Proposed solutions vetted at the kickoff meetings were then carried forward for implementation by the working groups. Key interventions that were agreed upon included: (1) the initiation of structured glucose monitoring for residents using CGM, requiring scanning of sensors 4 times per day to ensure a complete 24-hour glycemic profile; (2) the provision of staff education and training, by a diabetes expert, on CGM and use of the LibreView platform (Abbott Diabetes Care) for making clinical recommendations; and (3) the scheduling of interdisciplinary rounds as needed to optimize diabetes management, involving nursing staff, the clinical consultant pharmacist, and the dietician.

**Figure 1.**
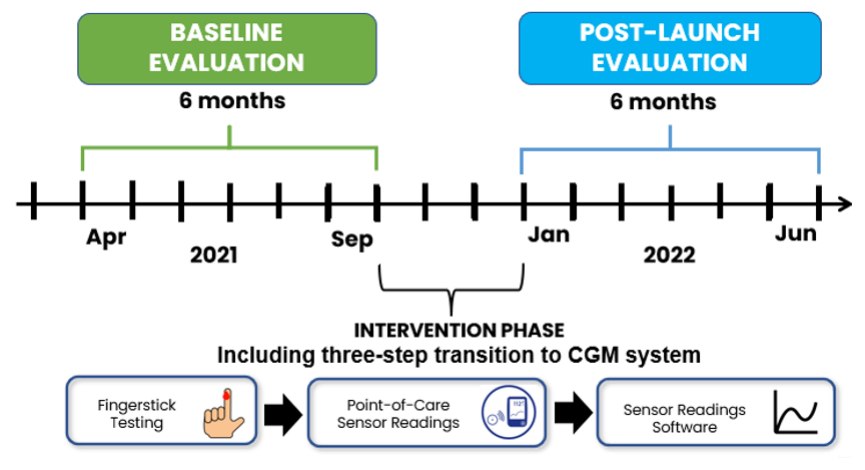
Project timeline including baseline evaluation, intervention phase, and post-launch evaluation. CGM: continuous glucose monitoring.

Over a 3-month period, system changes were gradually introduced following a stepwise approach to allow each facility to transition to CGM monitoring while addressing perceived risk associated with adoption of the new practice model. LTC homes progressed from traditional fingerstick testing to point-of-care sensor readings (where the reader was used simply to check readings at a point in time) and then to the full use of the software platform (Figure 1). All hypoglycemic readings detected by a sensor at a point-of-care test were validated using a fingerstick test.

### CGM Technology Used

The CGM sensor used was FreeStyle Libre 2 (Abbott Diabetes Care), with scans conducted using the FreeStyle Libre reader. One reader per resident was needed for scanning the sensors, which was carried out 4 times per day. Readers were then connected to a computer to upload data to the LibreView platform.

### Post-Launch Evaluation

#### Overview

Following implementation of the agreed changes, a 6-month evaluation was conducted to determine the sustained impact of the intervention. During this time, 35,971 glucose readings were gathered to evaluate changes in glucose management. Nursing staff were surveyed again to measure changes in nursing workload.

#### Nursing Workload

Nursing workload reduced from a mean (SD) of 5.1 (1.4) minutes per test to 3.1 (1.7) minutes per test, resulting in a 40% reduction in nursing time for an equivalent number of tests. This was estimated from survey results completed by over 60% of nursing staff.

#### Hypoglycemic Events

**Figure 2.**
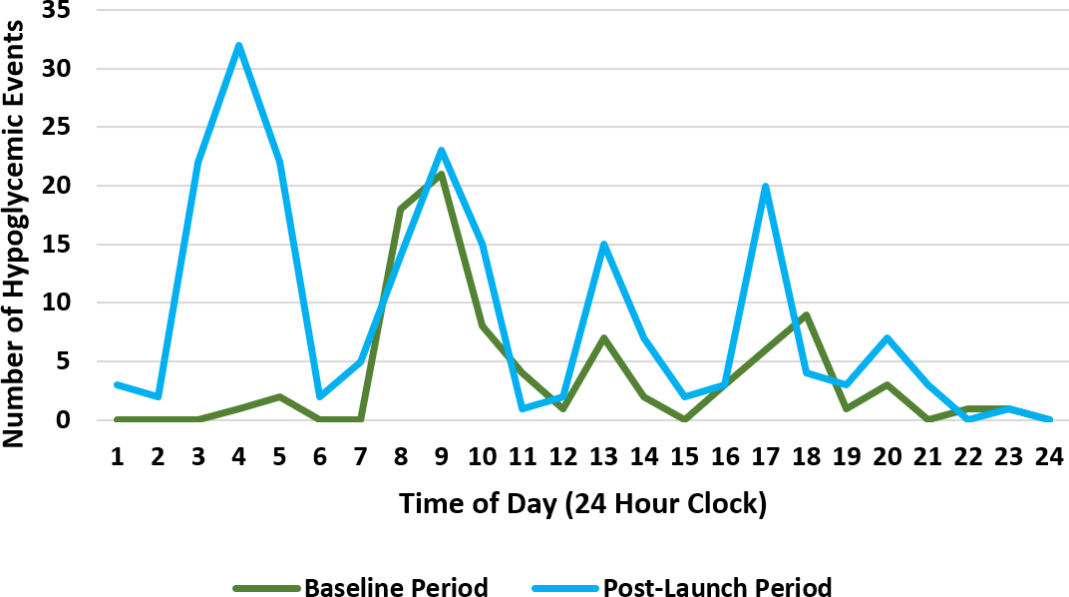
Hypoglycemic events detected at baseline versus post-launch evaluation by the time of day.

Based on the aggregate findings from the 2 participating LTC homes, point-of-care scans increased from 19,438 glucose readings in the 6-month baseline evaluation period to 35,971 glucose readings in the 6-month post-launch evaluation period, showing an increased frequency of monitoring. The number of hypoglycemic events detected (defined as glucose readings ≤4 mmol/L) increased from 88 (baseline) to 208 (post-launch evaluation) using CGM as a point-of-care test (Figure 2).

Repeat testing using a fingerstick device was performed for all observed hypoglycemic events, representing 0.6% (208/35,971) of all post-launch readings. The analysis of glucose data using the LibreView glucose monitoring software identified 1049 hypoglycemic events during the same post-launch period, representing a 12-fold increase in the number of detected events compared with the baseline period (Table 2).

**Table 2.** Number of hypoglycemic events detected at baseline versus the post-launch evaluation according to the screening method used.

Assessment period and screening method used	Number of hypoglycemic events
Baseline	
Blood Glucose Testing	88
Post-launch period	
Sensor reading only	208
Sensor reading software (Libreview)	1049

## Discussion

### Lessons Learned

This quality improvement initiative has shown that CGM can be successfully adopted in an LTC setting, reducing the amount of nursing time required per test. Importantly, there is an expected positive effect on residents’ quality of life by using sensor scans to reduce invasive fingerstick testing by over 99%.

### Importance of Previously Undetected Hypoglycemia

By capturing a 24-hour glycemic profile, this quality improvement monitoring strategy uncovered approximately 800 hypoglycemic events that would not have been detected using point-of-care scanning. These previously undetected hypoglycemic events occurred predominantly in the early morning hours when conventional blood glucose testing is difficult to perform with sleeping residents. Although these hypoglycemic events did not require rescue measures, the inherent risk associated with recurrent asymptomatic hypoglycemia is incontrovertible. The findings of this quality improvement initiative are in keeping with current research completed in older adults showing that at least 46% of hypoglycemic events happen overnight [23,27]. Using CGM software to understand the onset and duration of these hypoglycemic events can inform efforts to both reduce glycemic variability and increase time in range [9,21,22]. Clinical research has shown that raising HbA_1c_ targets in older patients, although recommended, does not obviate the risk of hypoglycemic events [22,28]. While HbA_1c_ continues to serve as a gold standard for assessing glycemic control, it does not confirm glycemic variability or the frequency of hypoglycemic events [29]. The time in range and time below range calculated by the CGM software can better measure the risk of hypoglycemic events [1,30].

### Impact on Nursing Workload and Resident Quality of Life

By saving 2 minutes per test with an average of 1030 tests per resident per year (the testing frequency in the 6-month baseline evaluation period), approximately 34 hours of nursing time could be saved per resident per year. At US $38.43 per hour, this would result in potential annual savings of nursing costs of over US $1306.45 per resident per year. While this is a simplified estimate that does not represent a formal health economic analysis or account for variability in wages, indirect costs, or other system-level considerations, time savings shown through this project nevertheless support the growing evidence that CGM can reduce costs associated with diabetes management outside of the hospital [31]. At the time of this initiative, it was necessary to tether the CGM reader device to a computer to upload data. More recently, the ability to use mobile device apps to scan CGM sensors and automatically upload data may mean that greater time savings can be achieved.

In LTC, full provincial coverage is available in Ontario for CGM readers and sensors for residents using insulin, as well as blood glucose test strips and readers. However, lancing devices and related supplies remain an ongoing cost incurred by LTC homes. While CGM is comparable in cost to intensive blood glucose monitoring, four or more blood glucose checks daily over an extended period is often impractical in LTC, so average traditional monitoring costs tend to be lower in practice. This initiative showed that there is a clear need for greater vigilance around glycemic variability, and demonstrated how CGM can make intensive glucose monitoring feasible. During the study period, CGM use allowed a larger number of point-of-care glucose tests to be conducted in a given amount of nursing time compared with fingerstick testing. Further use of the data uploaded to LibreView may allow intensive glucose monitoring to take place alongside reductions in nursing workload. The number of hypoglycemic events detected during this project highlights that, even when CGM technology is not available, periodic intensive glucose monitoring using fingerstick testing should be considered.

### Concerns About the Use of CGM Technology for Residents Living With Cognitive Impairment

For residents living with cognitive impairment, reducing fingerstick tests would be beneficial, as they may cause distress and reduce well-being among people who do not understand the purpose of the tests [32]. However, one concern expressed in advance of this project was that residents living with cognitive impairment might remove the sensors if they did not understand their purpose. Although this was not measured as an outcome of this pilot study, staff did not report sensor removal to be a major issue, and any problems of this sort that made glucose checking more time consuming would have been captured within the staff survey.

### Overcoming Concerns Regarding Accuracy and Reliability of CGM Readings

While CGM has the potential to reduce hypoglycemia and improve time in range, user acceptance remains a critical component for translating clinical findings into meaningful changes in therapy [33]. Numerous studies have attempted to validate CGM systems by comparing sensor readings to concurrent capillary blood draws [34,35]. These comparisons typically report analytical performance in terms of a MARD using these paired measures. The average discrepancy observed between glucose readings performed by a device under review and capillary glucose measures as the gold standard offers an intuitive method to compare the performance of different glucose monitoring systems. The top seven highest-performing fingerstick glucometers have MARDs between 5.6% and 8.2% versus reference measurements, compared with 9.2% for FreeStyle Libre 2 and 12.8% for another CGM system [36-38]. It is important not to overemphasize differences in MARD but consider whether the differences are clinically significant. In the case of a device with a MARD of 12% being compared with a device with a MARD of 6%, for a capillary blood glucose reading of 4 mmol/L, the first device would on average generate a reading between 3.5 mmol/L and 4.5 mmol/L, while the second device would on average generate a reading between 3.8 mmol/L and 4.2 mmol/L. It is unlikely that the wider range of values of the former device would alter how this clinical situation is handled, particularly if coupled with a trend arrow. Error grids such as the consensus error grid are another way to infer the clinical relevance of these ranges [39]. A study of a CGM system with a MARD of 12.8% showed that 98.7% of values fell within the preferred Zones A and B [38]. In the context of managing medication regimens in LTC, this would suggest that CGM is sufficiently accurate. Regardless, our project demonstrated that ongoing concerns regarding meter accuracy can be effectively addressed using a fingerstick confirmation in cases where there is a low glucose reading or a suspected hypoglycemic event. This requires a fingerstick test in fewer than 1% of sensor readings. Rather than relying on a single system or value, combining both CGM and fingerstick monitoring allowed for a more complete and clinically useful picture of glucose control. This integrated approach supported more confident decision-making, particularly when interpreting outlier readings or managing suspected hypoglycemia.

The trend arrow provided by the CGM reader was new to many staff, who were accustomed to the point-in-time results obtained from fingerstick testing. Because part of the concern around CGM accuracy was based on the delay between readings taken from blood and those from interstitial fluid (for the most recent generation of CGM sensors, the mean lag in readings is 1.8 min vs venous blood glucose [40]), the trend arrow was found to be helpful. However, as noted above, the electronic health record system used did not include information on whether trend arrows were used to guide decisions.

### Future Directions

This pilot project has shown that transitioning to CGM use in LTC is possible and can substantially reduce the nursing time needed for glucose monitoring. By the end of the study period, the 2 participating LTC facilities had switched to using CGM for point-of-care scans, with some use of glucose trend arrows (although this could not be measured directly), and were beginning to make use of the LibreView platform. As CGM enables more frequent and sensitive glucose monitoring, there is a potential risk of overtreatment. Careful interpretation of CGM data within the context of each resident’s clinical situation will be essential to avoid unnecessary interventions. At this stage, CGM was still largely being used as a direct replacement for fingerstick testing (ie, as a method of checking residents’ glucose levels before administering insulin). Future research will investigate how the detailed data captured by the CGM system can translate into better diabetes management, including potentially medication changes and, where appropriate, deprescribing, in the LTC setting. Furthermore, the study did not formally capture data on sensor wear time, dislodgements, or skin reactions, factors that are particularly relevant to evaluating feasibility in cognitively impaired residents. Collecting such data in future studies could strengthen the assessment of real-world usability and clinical integration.

## Conclusions

The introduction of more intensive monitoring using CGM in LTC can significantly improve the detection of hypoglycemic events while reducing nursing workload. Future studies are needed to quantify the impact on health outcomes including quality of life measures. Opportunities to further reduce nursing workload and improve care may exist through further advances in the use of CGM technology and potential coverage for residents on non-insulin treatment regimens.

## Abbreviations

CGM: continuous glucose monitoring
HbA_1c_: glycated hemoglobin
LTC: long-term care
MARD: mean absolute relative difference
